# ACLY alleviates cerebral ischemia/reperfusion injury by reducing oxidative stress and enhancing mitochondrial function via histone acetylation

**DOI:** 10.1038/s41419-026-09021-4

**Published:** 2026-06-22

**Authors:** Xiaona Sun, Rui Zhao, Qidi Zhou, Xiaorong Wang, Yuxin Li, Yongkun Yang, Zhiyuan Zhao, Yu Cui, Rui Xu

**Affiliations:** 1 Department of Interventional Radiology, The Affiliated Hospital of Qingdao University, 16 Jiangsu Road, Qingdao, 266000 Shandong China; 2 China Uruguay Bio-nano Pharmaceutical Joint Laboratory, Qingdao University, Ningxia Road 308, Qingdao, 266071 Shandong China; 3 Qingdao Medical College, Qingdao University, Ningxia Road 308, Qingdao, 266071 Shandong China; 4 Department of Department of Neurosurgery, The Affiliated Hospital of Qingdao University, 16 Jiangsu Road, Qingdao, 266000 Shandong China

**Keywords:** Stroke, Cellular neuroscience

## Abstract

Cell metabolism and epigenetic regulation play crucial roles in modulating cerebral ischemia/reperfusion (I/R) injury. How cell metabolism regulates cerebral I/R injury by regulating epigenetic modifications remains unclear. In this study, we utilized an in vivo injury model of transient middle cerebral artery occlusion (tMCAO) in C57BL/6 mice. The middle cerebral artery was occluded for 90 min, followed by reperfusion at different time points. We observed that the expression of ATP-citrate lyase (ACLY), an important enzyme involved in lipid synthesis, was significantly upregulated under cerebral I/R conditions. Inhibition of ACLY markedly exacerbated cerebral I/R injury in vivo. ACLY inhibition and knockdown in vitro also reduced cell viability in cultured neurons following oxygen-glucose deprivation/reoxygenation (OGD/R). Mechanistic studies revealed that ACLY enhances histone acetylation at the promoter regions of mitochondrial respiratory chain complexes by facilitating the accumulation of acetyl-CoA, thereby improving mitochondrial function and attenuating oxidative stress. Our findings reveal a novel metabolic-epigenetic axis mediated by ACLY in the regulation of cerebral I/R injury which may serve as a potential target for therapeutic intervention in ischemic stroke.

**Figure:**
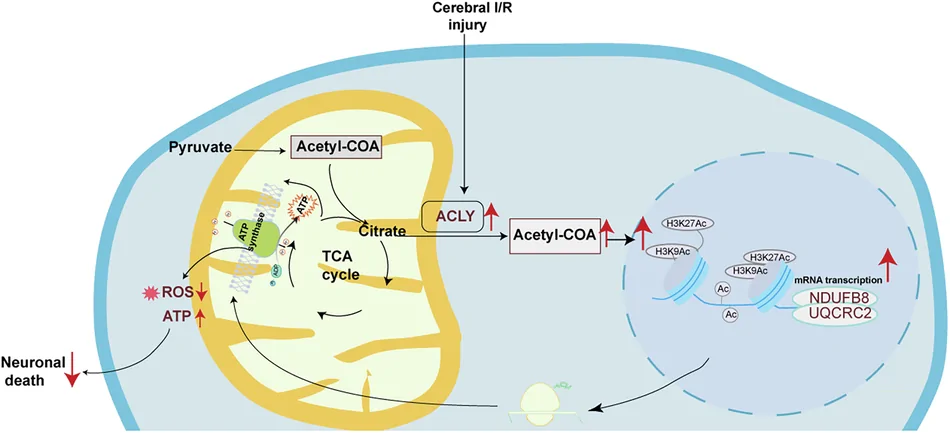

## Introduction

Ischemic stroke is characterized by high morbidity, high disability rate, and high mortality, imposing a tremendous burden on patients and society [1, 2]. Currently, the most effective therapeutic interventions for ischemic stroke is mechanical thrombectomy or thrombolytic therapy with recombinant tissue plasminogen activator (rt-PA) [3], often accompanied by adjuvant approaches such as ischemic preconditioning, antioxidants, and anti-inflammatory medications [4–7]. Although reperfusion therapy partially restores blood flow during the acute phase, secondary injuries may exacerbate ischemic pathology [8]. Thus, there is an urgent requirement to develop novel and alternative cytoprotective strategies to mitigate cerebral ischemia-reperfusion injury, improve patient outcomes, and enhance the efficacy of existing clinical interventions.

Fatty acid metabolism has recently emerged as a potential energy reserve after stroke and plays important roles in both the pathogenesis and progression of cerebrovascular injury [9, 10]. Lipids are essential for maintaining the structural integrity of brain cells, influencing membrane fluidity and permeability, facilitating energy metabolism, and regulating various signaling pathways [11–13]. Accumulating evidence indicates that several lipid metabolism-related enzymes have been reported to participate in cerebral I/R injury. For instance, acyl-CoA synthetase long chain family member 4 (ACSL4) has been revealed to exacerbate I/R injury by facilitating neuronal ferroptosis [14]. Inhibition lipogenesis through targeting fatty acid synthase (FASN) impairs functional recovery after I/R injury [15]. Despite these advances, the roles of other lipid metabolic enzymes and specific lipid species in stroke pathology remain to be fully elucidated and warrant further investigation.

ATP-citrate lyase (ACLY), localized in both the cytoplasm and nucleus, is a key metabolic enzyme involved in fatty acid synthesis by catalyzing the conversion of mitochondrially-derived citrate into acetyl-coenzyme A (CoA) [16, 17]. Cytosolic acetyl-CoA primarily serves as a precursor for fatty acid synthesis, while nuclear acetyl-CoA functions as an acetyl donor for histone acetylation [18, 19], thereby linking cellular metabolism to epigenetic regulation [20, 21]. ACLY have been implicated in regulating various metabolic and pathological conditions, such as liver, cardiovascular, and neurological disorders [22, 23]. Inhibition of ACLY has been shown to reduce α-tubulin acetylation, leading to compromised microtubule stability and impaired cognitive function [22]. In addition, ACLY modulates the acetylation of smad2/3 in the pathogenesis of Duchenne muscular dystrophy [24] and protects the liver against ischemia-reperfusion injury via nuclear translocation [18]. Despite these advances, the specific role of ACLY in mediating cerebral I/R injury remains unexplored.

In this study, we observed upregulation of ACLY in neurons on cerebral I/R injury. Inhibition of ACLY significantly exacerbated neuronal death both in vivo and in vitro. Mechanistically, ACLY promotes acetyl-CoA production, which in turn enhances histone acetylation modification levels in the promoter regions of mitochondrial complexes I and III and facilitates their transcription, thereby reducing mitochondrial ROS, and improve mitochondrial function to exert neuroprotective effects.

## Materials and methods

### Animals and focal cerebral ischemia

Adult male C58BL6 mice were purchased from Jinan Pengyue Laboratory Animal Breeding Co., Ltd.

Buprenorphine (0.05 mg/kg, subcutaneous) for analgesia was given 1 h before the surgery. Male C57BL/6 mice (9–12 weeks old, 20–25 g) were anesthetized with 4% isoflurane by using a precision isoflurane vaporizer, an induction chamber, and a nose-cone delivery system (RWD, SuperV, and ZH-MZJ). Transient focal cerebral ischemia was induced using the intraluminal suture occlusion method. Briefly, the cervical region was aseptically prepared, and a midline skin incision was made to expose the left external carotid artery (ECA). A monofilament nylon suture (L1800, Jialing, China) was then inserted into the ECA and advanced through the carotid artery to occlude the origin of the middle cerebral artery (MCA) for 90 min. For the sham group, mice received the same surgical procedures without filament insertion. To restore the MCA blood flow, the suture was gently pulled out. The stump of the ECA was ligated, and the surgical wounds were closed. Regional cerebral blood flow (rCBF) was monitored in real-time using laser Doppler flowmetry (PeriCam PSI Z) to verify successful vascular occlusion and subsequent reperfusion. Normothermia was maintained throughout the surgical and recovery periods. Mice were maintained on a heating pad (RWD, 69003) throughout the procedure, and their respiration was continuously monitored using a respiratory monitoring system. During the recovery phase, animals were placed in a warmed cage with free access to food and water. Twelve hours later, Buprenorphine was given again. A total of 225 mice were subjected to tMCAO, among which 30 mice died within 24 h after reperfusion and were excluded from the final data analysis. Sample size was determined with reference to previous studies and practical considerations [25, 26],

To accurately assess the results, animals were randomly assigned to experimental groups using a predefined randomization table. Investigator A performed all MCAO and sham surgeries and was not involved in treatment allocation. Investigator B was responsible for group assignment and drug administration according to the predefined randomization scheme and was aware of the treatment allocation. Outcome assessments, including neurological evaluation, TTC staining, image acquisition and data analysis were performed by Investigator A, who was blinded to group allocation and not involved in treatment administration. Group identities were only revealed after completion of statistical analysis. All experimental procedures were conducted in accordance with ARRIVE 2.0 and STAR guidelines.

### TTC staining and infarct volume measurement

At 24 h after tMCAO, the animals were anesthetized with CO_2_ and perfused with PBS. Brains were harvested for 2,3,5-triphenyltetrazolium chloride (TTC) (T8877, Sigma Aldrich) staining to evaluate infract volume. Briefly, the excised brains were immediately placed at –80 °C for 15 min, then coronally sectioned into 1.2 mm thick slices, which were incubated with 2% TTC in phosphate-buffered saline (PBS) at 37 °C for 30 min under light-protected conditions. Post-staining, slices were fixed overnight at 4 °C in 4% paraformaldehyde (PFA). Viable brain tissue was stained a deep vermilion, while infarcted regions remained unstained (appearing pale white) [27]. Stained sections were digitally scanned, and infarct volume was quantified using ImageJ software (NIH, USA) following established protocols [26]. Infract volume was adjusted based on the ipsilateral edema size in compliance with “Swanson’s correction [28]”. The adjusted infarct volume was then normalized to the contralateral hemisphere volume to account for potential artifacts caused by tissue processing, including swelling or shrinkage.

### CCK8 assay

To assay cell viability, Cell Counting Kit-8 (TargetMol) was employed following the manufacturer’s protocol. Cortical neurons were plated in 48-well plates at a density of 3.5 × 10^5^ cells per well and subjected to siRNA transfection or drug intervention. After 24 h of re-oxygenation, CCK-8 solution was added to each well in an incubator at 37 °C for 4–6 h. The OD value was measured at 450 nm by SpectraMax ABS (Molecular Devices, USA) to assess cell viability. A blank control was included to subtract background absorbance. Cell viability was calculated as:

$${\rm{Cell\; viability}}( \% {\rm{of\; control}})=\frac{{{\rm{OD}}}_{\mathrm{treated}}-{{\rm{OD}}}_{{\rm{blanked}}}}{{{\rm{OD}}}_{\mathrm{control}}-{{\rm{OD}}}_{{\rm{blanked}}}}\times 100 \%$$

### Cell culture and siRNA-mediated interference

To suppress ACLY expression, neurons were transfected with 20 nM siRNA targeting ACLY using the Kermey transfection reagent (MLR-0201) in accordance with the manufacturer’s protocol. Following a 48-h incubation period to allow for optimal gene silencing, the transfected neurons were treated with OGD/R and then were harvested for further experiments. The siRNA sequences utilized were as follows: Mouse ACLY Sense: 5′-UGAAUACCGAGGACAUUAAtt-3′; Mouse ACLY Antisense: 5′-UUAAUGUCCUCGGUAUUCAtt-3′.

### Mitochondrial ROS staining and mitochondrial membrane potential assay

Mitochondrial ROS were detected using the MitoSOX Red (M36008, Invitrogen). Mitochondrial Membrane Potential Assay was measured using the TMRE (C2001S, Beyotime). PBS-washed cells were incubated with 2.5 µM MitoSOX for 20 min and 500 nM TMRE (C2001S, Beyotime) for 40 min at 37 °C. The staining was viewed by a confocal scanning microscope (Nikon-Ti2-E).

### Statistical analysis

All experiments were conducted using randomized group allocation and blinded assessment procedures. Statistical analyses were performed using GraphPad Prism software. Power analysis was performed to ensure the study had sufficient statistical power to detect significant effects confidently. The normality of data distribution was assessed using the Shapiro-Wilk test for each group. For normally distributed data, one-way or two-way ANOVA followed by Bonferroni’s or Tukey’s test was used for multiple comparisons. Data are presented as means ± S.D. and represent at least five independent experiments. The Kruskal–Wallis test was used to compare multiple groups that are not normally distributed. Statistical significance was defined at *p* < 0.05.

## Results

### The expression of ACLY is increased in neurons after cerebral I/R injury

**Fig. 1:**
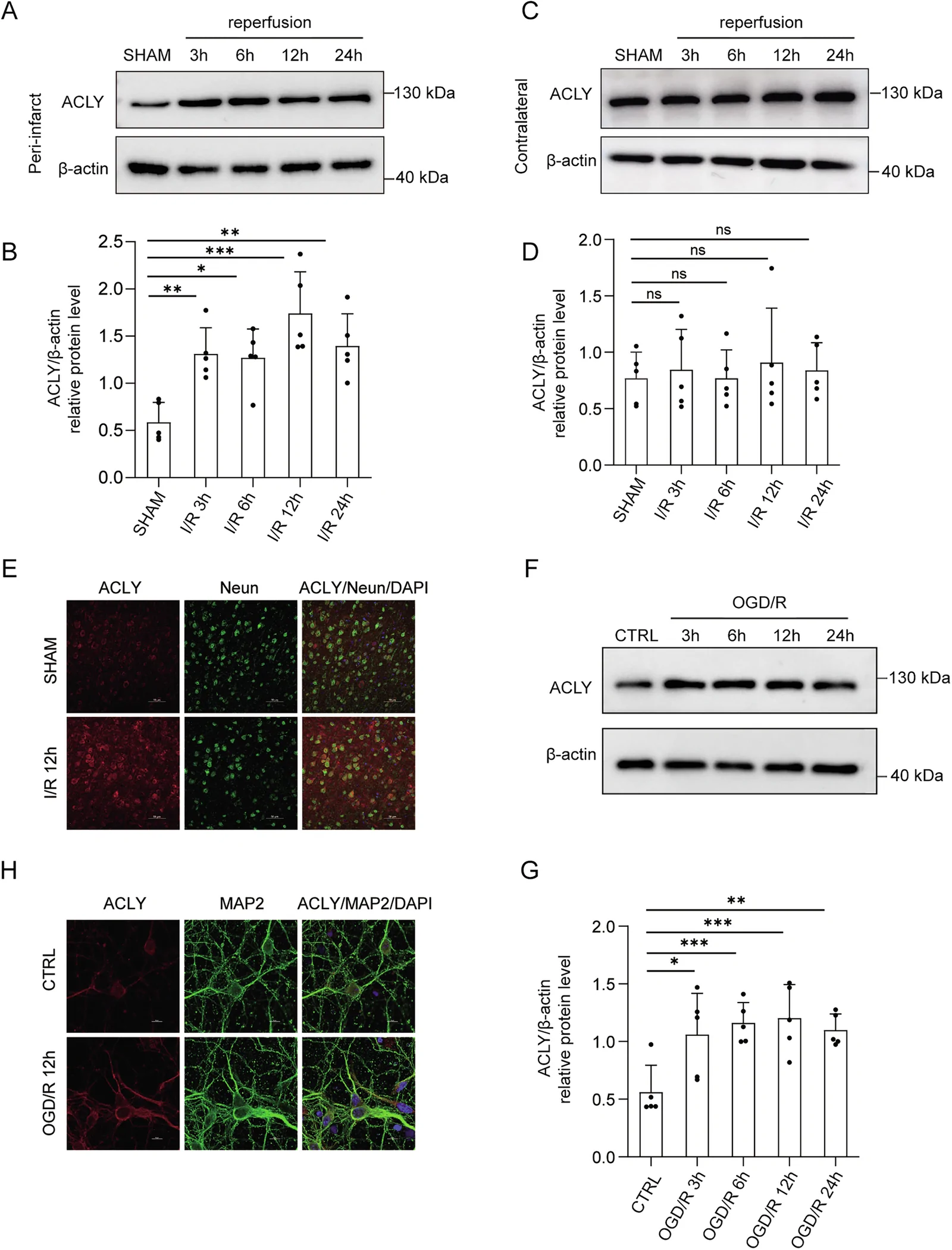
ACLY expression is upregulated after cerebral I/R injury. Immunoblot images (**A**) and quantification (**B**) of ACLY protein levels in tissue extracts from the penumbra region of mouse brains after 90-min tMCAO followed by different reperfusion periods (*n* = 5). β-actin as a loading control. Immunoblot images (**C**) and quantification (**D**) of ACLY protein levels in tissue extracts from the contralateral region (*n* = 5). **E** Confocal microscopy images of brain slices from the ischemic penumbra in different groups were stained with Neun (green) and ACLY (red), Scale bar = 50 μm. Immunoblot images (**F**) and quantification (**G**) of ACLY in cultured primary cortical neurons by OGD treatment for 1.5 h and reoxygenation at different time groups (*n* = 5). **H** Confocal microscopy images of ACLY (red) and MAP2 (green) immunofluorescence staining in primary cortical neurons by OGD treatment for 1.5 h and reperfusion for 12 h. Scale bar = 10 μm. Data are expressed as means ± S.D., for all panels: *P < 0.05, **P < 0.01, ***P < 0.001, ns stands for no significance by one-way-ANOVA analysis with Tukey’s Multiple Comparison Test applied afterward (**B**, **D**, **G**).

To investigate the role of ACLY in brain ischemia-reperfusion injury, we subjected mice to transient middle cerebral artery occlusion (tMCAO) and examined the expression of ACLY in the cerebral cortex at various reperfusion time points using Western blotting (WB). The results revealed that ACLY expression progressively increased in the peri-infarct region following I/R, while remaining unchanged in the contralateral hemisphere (Fig. 1A–D). To further identify the cell types involved in elevated ACLY expression in the brain, we prepared frozen sections of mouse brain tissues and performed immunofluorescence staining. The results showed that ACLY was mainly colocalized with neurons (NeuN^+^) in sham-operated cortex, and its expression was significantly elevated at 12 h post-reperfusion, consistent with WB findings (Fig. 1E). Thus, ACLY expression is upregulated in neurons after cerebral I/R injury.

We further validated these observations in vitro by isolating and culturing primary cortical neurons, then subjecting them to oxygen-glucose deprivation/reperfusion (OGD/R). Similar to the in vivo results, OGD/R treatment led to a significant increase in ACLY expression (Fig. 1F, G). Immunofluorescence analysis confirmed that ACLY was primarily localized in the neuronal cytoplasm, with markedly increased fluorescence intensity post-OGD/R, further supporting its upregulation (Fig. 1H). Collectively, these findings suggest that ACLY expression is significantly upregulated in neurons following brain I/R injury.

### ACLY inhibition exacerbates brain ischemia–reperfusion injury

**Fig. 2:**
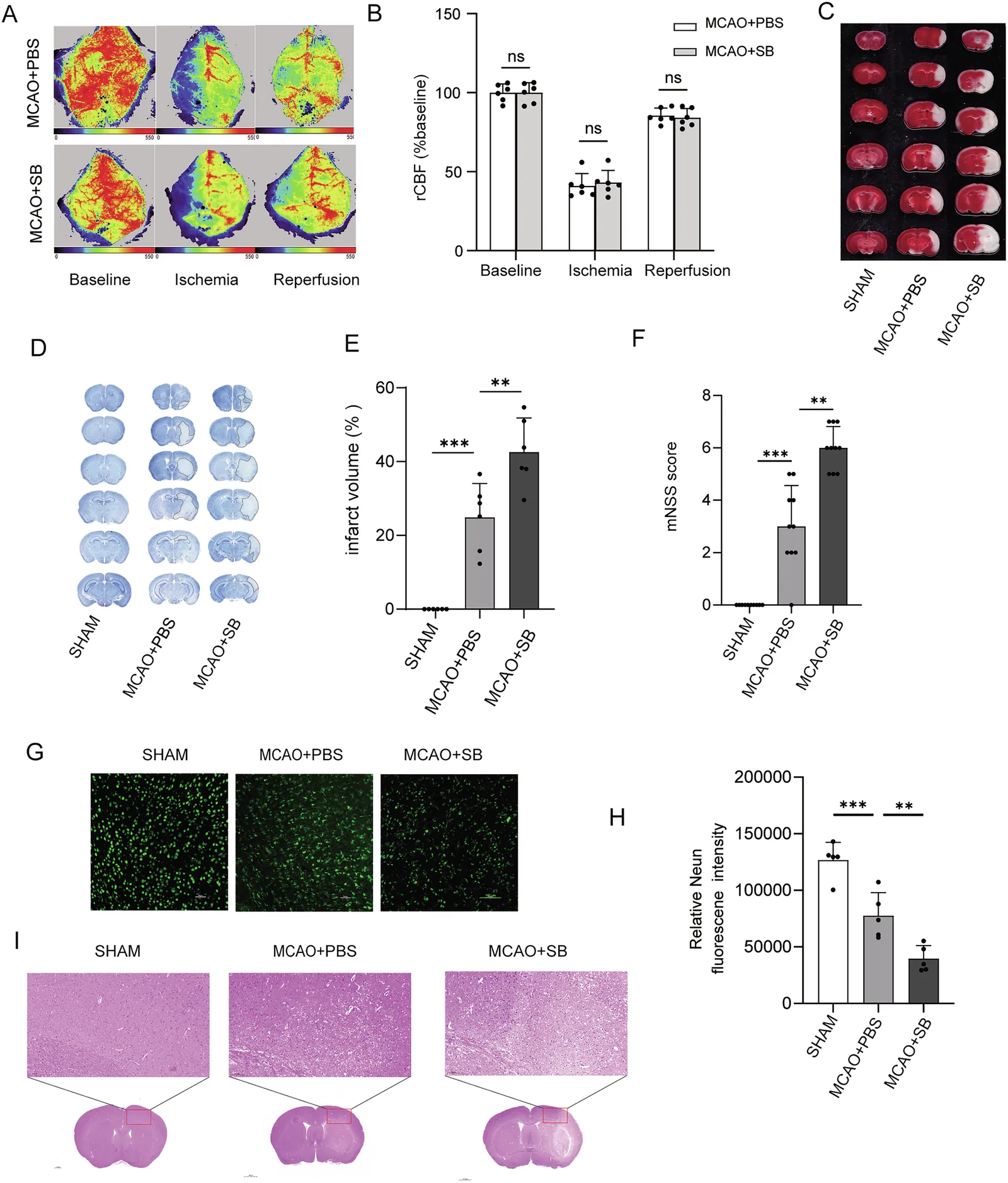
ACLY inhibition aggravates cerebral I/R Injury. **A** Regional cerebral blood flow (rCBF) was imaged at different time periods, color changes from red to blue indicates a decrease in blood flow. **B** Statistical comparison of rCBF at different time periods between SB204990(SB)-treated and control groups, (*n* = 6). The data shown are the percentage of rCBF compared with the baseline. The test of “baseline” “ischemia” “reperfusion” separately represents 15 min before tMCAO, after tMCAO and 15 min after reperfusion. **C** Representative brain slices stained by TTC of infarct volume at 24 h after tMCAO, (*n* = 6 for each treatment). **D**, **E** Representative brain slices stained by Nissl (**E**) and quantification (**F**) of infarct volume at 24 h after tMCAO. (*n* = 6 for each treatment). **F** Evaluation of neurological deficits at 24 h following reperfusion (*n* = 10). Confocal microscopy images (**G**) and quantification (**H**) of fluorescence intensity brain slices from the ischemic penumbra in different groups were stained with NeuN (green) at 24 h after reperfusion. *n* = 5 animals per group, Scale bar = 100 μm. **I** Representative HE staining sections at 24 h after reperfusion. Data are expressed as means ± S.D., for all panels: *P < 0.05, **P < 0.01, ***P < 0.001, ns stands for no significance by two-way-ANOVA analysis with Bonferroni Test (**B**) applied afterward and one-way-ANOVA analysis with Tukey’s Multiple Comparison Test applied afterward (**E**, **F**, **H**).

To investigate the effects of ACLY on brain ischemia-reperfusion (I/R) injury, we established tMCAO model on mice and administered SB204990, a specific inhibitor of ACLY, or PBS solution. Successful induction of the I/R model was confirmed using relative cerebral blood flow (rCBF) measurements and TTC staining. During both the ischemic phase and reperfusion, rCBF remained comparable between the ACLY inhibitor group and control groups, indicating no significant influence on blood flow after ACLY inhibition (Fig. 2A, B). Importantly, the inhibitor group exhibited a significantly larger brain infarct volume at 24 h after reperfusion compared to the controls as determined by TTC staining (Fig. 2C, Fig. S1A) and Nissl staining (Fig. 2D, E). Furthermore, neurological deficits assessed by the mNSS were significantly worse in the inhibitor-treated group following I/R injury (Fig. 2F).

To further examine the impact of SB204990 on neurons post-I/R injury, immunofluorescence staining with anti-NeuN antibody was performed on frozen brain sections. SB204990 exacerbated neuronal death in mouse brain tissues following I/R injury (Fig. 2G, H). Consistent with these findings, HE revealed a marked matrix loosening, edema, neuronal atrophy and nuclear atrophy in the SB204990-treated group after I/R injury (Fig. 2I), indicative of impaired neuronal integrity and protein synthesis. These results suggest that inhibition of ACLY by SB204990 exacerbates cerebral I/R injury in mice.

### Knockdown of ACLY increased neuronal death after OGD/R treatment

**Fig. 3:**
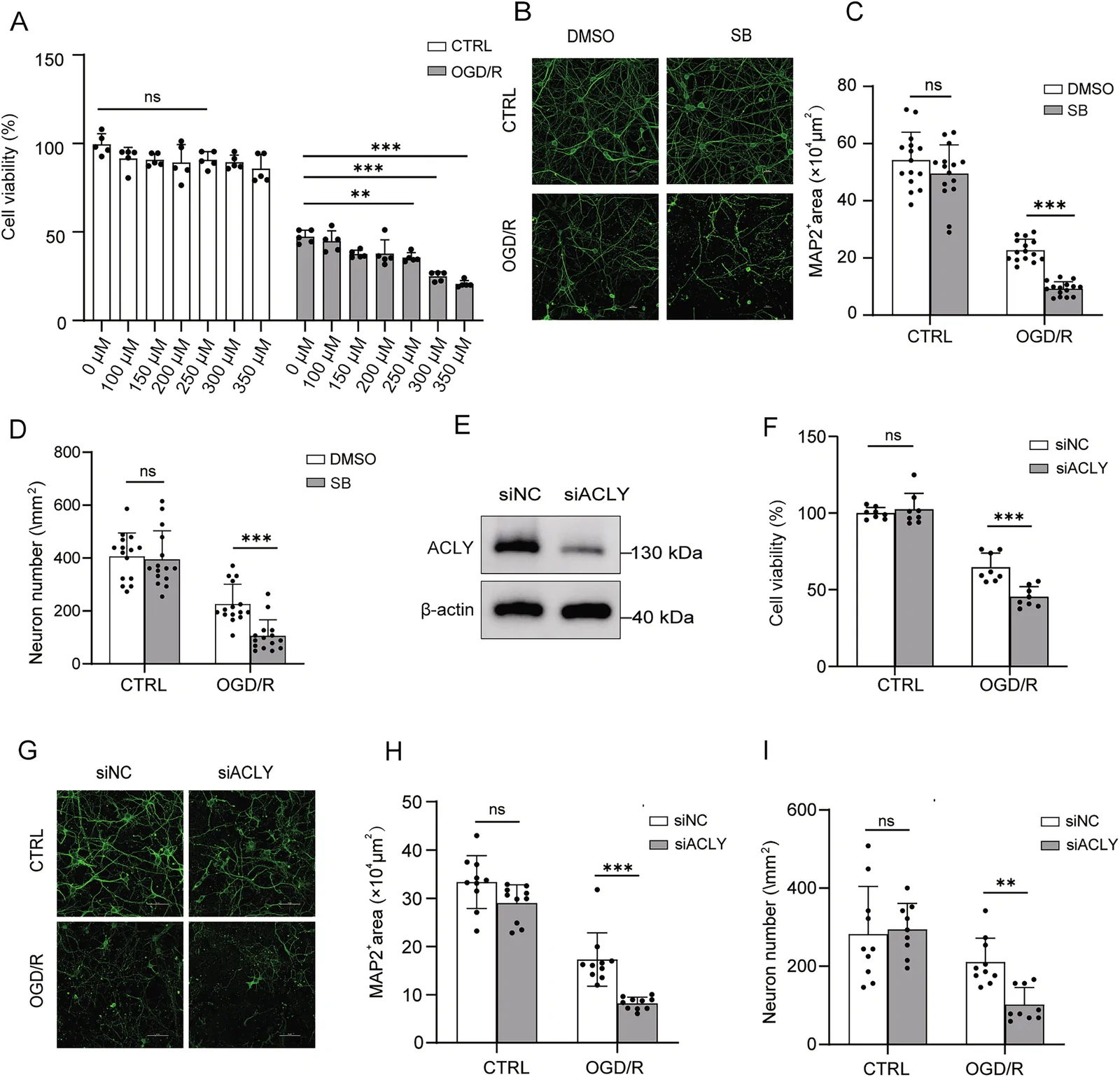
Knockdown of ACLY significantly exacerbated neuronal death after OGD/R treatment. **A** The primary cultured cortical neurons were subjected to OGD treatment for 2.5 h and reoxygenated with SB204990 for 24 h. Cell viability was evaluated, (*n* = 5). Representative MAP2 area and neuronal number (**B**) and statistical analysis (**C**, **D**) in primary cortical neurons subjected to OGD treatment for 2 h and reoxygenation with SB204990 for 24 h, scale bar = 20 μm, (*n* = 5). **E** Representative immunoblot of primary cultured cortical neurons transfected with siNC and siACLY. **F** The primary cultured cortical neurons were first transfected with siNC and siACLY for 3 days and then subjected to OGD treatment for 2 h and reoxygenation for 24 h. Cell viability was evaluated, *n* = 5. Representative MAP2 area and neuronal number (**G**) and statistical analysis (**H**, **I**) of primary cultured cortical neurons transfected with siNC and siACLY. scale bar = 20 μm (*n* = 5). Data are expressed as means ± S.D., for all panels: *P < 0.05, **P < 0.01, ***P < 0.001, ns stands for no significance by two-way-ANOVA analysis followed by Bonferroni Test.

We further investigated whether ACLY inhibition exacerbated brain infarction by directly affecting neurons using the OGD/R system in vitro. Cultured cortical neurons were treated with varying concentrations of SB204990 following reoxygenation or under normal conditions, and neuronal viability was assessed. CCK8 assay demonstrated that SB204990 decreased neuronal viability in a concentration-dependent manner after OGD/R, with statistically significant reductions observed at 250 μM. In contrast, SB204990 treatment had no effect on neuronal viability under normal conditions (Fig. 3A). Further immunofluorescence staining with anti-MAP2 antibodies revealed that ACLY inhibitions resulted in reduced neuronal numbers, decreased MAP2 expression, and more severe dendrites fragmentation following OGD/R treatment [29] (Fig. 3B–D).

To confirm the specific effects of ACLY inhibition on neuronal death, ACLY-targeting siRNA was used to knock down its expression on primary neurons and neuronal viability was examined. Western blot analysis confirmed efficient knockdown of ACLY in siACLY-transfected neurons compared to siNC controls (Fig. 3E). Consistent with pharmacological inhibition, genetic knockdown of ACLY also led to significantly reduced neuronal viability after OGD/R (Fig. 3F). These findings were corroborated by MAP2 immunostaining, which showed increased structural damage and diminished neuronal numbers in ACLY-knockdown neurons (Fig. 3G–I). Collectively, these findings suggest that ACLY inhibition or knockdown increases neuronal death after OGD/R treatment in vitro.

### ACLY reduces neuronal death by modulating mitochondrial ROS accumulation and function

**Fig. 4:**
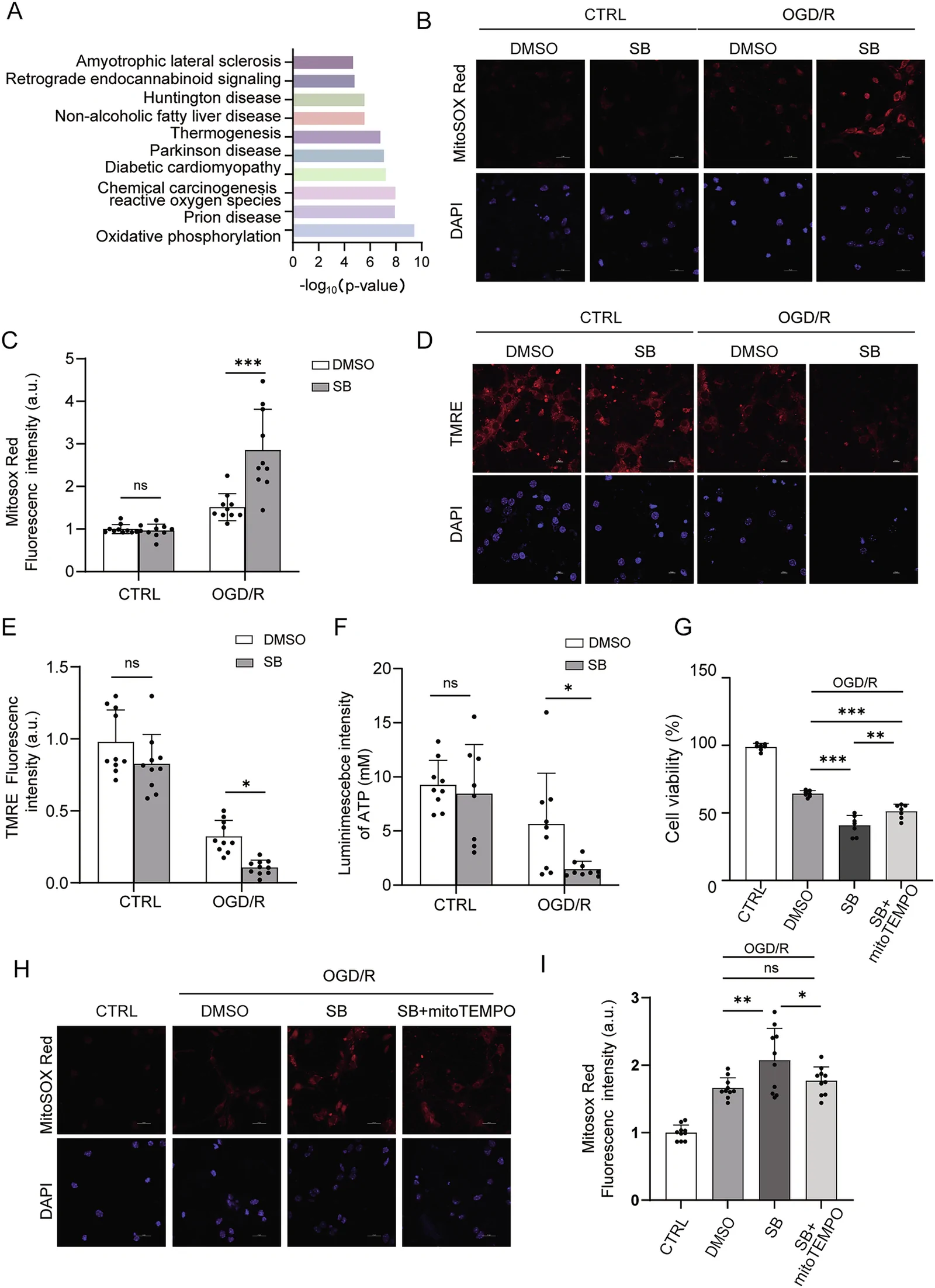
ACLY reduces neuronal death by modulating mitochondrial ROS accumulation and function. **A** KEGG pathway enrichment analysis of genes with significant changes in expression in neurons treated with DMSO or SB204990, based on RNA-seq results obtained at 12 h after OGD/R (*P* < 0.05). Representative MitoSOX immunofluorescence staining (**B**) and statistical analysis (**C**) in primary cortical neurons subjected to OGD treatment for 1.5 h and reoxygenation with SB204990 for 12 h, scale bar = 20 μm (*n* = 5). Representative TMRE immunofluorescence staining (**D**) and statistical analysis (**E**) in primary cortical neurons, scale bar = 10 μm (*n* = 5). **F** ATP release was assessed in neuronal cells subjected to OGD treatment for 3.5 h and reoxygenation with SB204990 for 24 h (*n* = 9). **G** Neuronal viability at 24 h after reperfusion measured by CCK8 assay. Representative MitoSOX immunofluorescence staining (**H**) and statistical analysis (**I**) in primary cortical neurons subjected to OGD treatment for 1.5 h and reoxygenation with SB204990 or MitoTEMPO for 12 h, scale bar = 20 μm (*n* = 5). Data are expressed as means ± S.D., for all panels: *P < 0.05, **P < 0.01, ***P < 0.001, ns stands for no significance by two-way-ANOVA analysis with Bonferroni Test (**C**, **E**, **F**) applied afterward and one-way-ANOVA analysis with Tukey’s Multiple Comparison Test applied afterward (**G**, **I**).

To elucidate the mechanism by which ACLY modulates neuronal survival after cerebral ischemia-reperfusion (I/R) injury, we employed SB204990-mediated ACLY inhibition in OGD/R-treated neuronal cells and performed comprehensive transcriptomic profiling. Kyoto Encyclopedia of Genes and Genomes (KEGG) enrichment analysis revealed that top three pathways affected by ACLY suppression were mainly metabolic pathways associated with mitochondrial function and reactive oxygen species (ROS) production (Fig. 4A). Subsequently, we investigated the impact of ACLY inhibition on mitochondria health. Immunofluorescence staining demonstrated that OGD/R treatment significantly elevated mitochondrial ROS levels in cortical neurons, an effect that was further amplified by SB204990-induced ACLY inhibition (Fig. 4B, C). Consistent with this, ACLY inhibition exacerbated the OGD/R-induced loss of mitochondrial membrane potential (ΔΨm), a critical indicator of mitochondrial integrity (Fig. 4D, E). Considering the central role of mitochondria in cellular energy metabolism, we assessed neuronal ATP levels and found that OGD/R markedly reduced ATP content compared to control conditions, with ACLY inhibition causing an additional decline in ATP production (Fig. 4F). These findings collectively demonstrate that ACLY inhibition impairs mitochondrial function after OGD/R treatment.

As reactive oxygen species (ROS) overproduction a well-established contributor to I/R-induced neuronal injury, we investigated whether the elevated ROS levels resulting from ACLY inhibition contribute to increased neuronal death. Supplementary of Mito-TEMPO, a recently characterized mitochondria-targeted antioxidant with potent superoxide-scavenging capacity [30], effectively suppressed mitochondrial ROS generation after ACLY inhibition (Fig. 4H–I). Importantly, co-treatment with Mito-TEMPO significantly rescued neuronal viability after ACLY inhibition in the context of OGD/R (Fig. 4G). These results suggest that ACLY inhibition increases neuronal death by promoting excessive ROS accumulation and impairing mitochondrial function.

### ACLY-inhibition promotes neuronal death by reducing histone acetylation

**Fig. 5:**
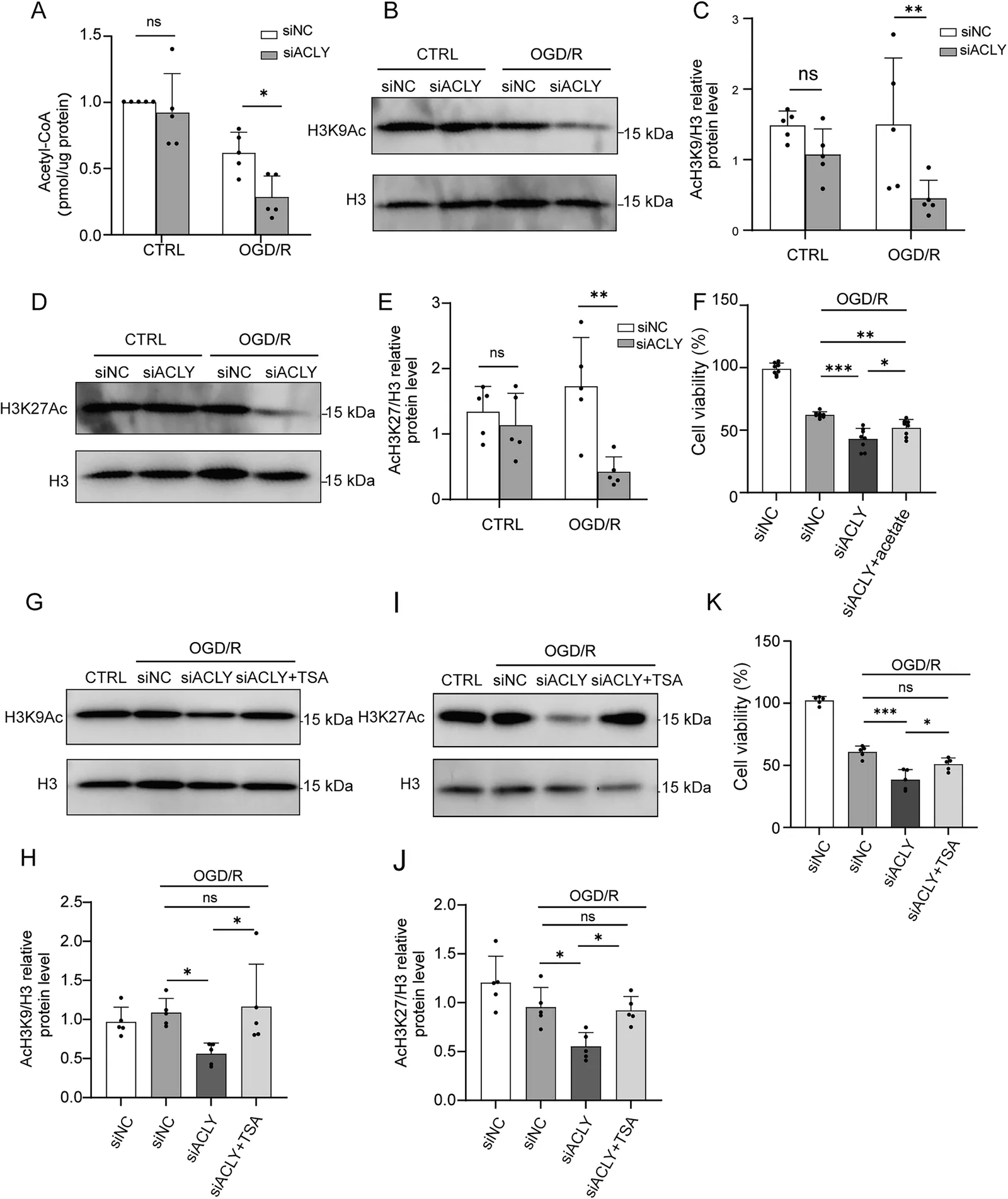
ACLY-inhibition promotes neuronal death via reducing histone acetylation. **A** ELISA analysis of acetyl-CoA levels in the supernatants from neuronal cells-transfected with siNC or siACLY followed by OGD/R treatment, (*n* = 5). Immunoblot images (**B**) and quantification (**C**) of H3K9ac protein levels in neurons- transfected with siNC or siACLY exposed to OGD treatment for 1.5 h and reoxygenation for 12 h. H3 as a loading control (*n* = 5). Immunoblot images (**D**) and quantification (**E**) of H3K27ac protein levels in neurons-transfected with siNC or siACLY exposed to OGD treatment for 1.5 h and reoxygenation for 12 h (*n* = 5). **F** The primary cultured cortical neurons were subjected to OGD treatment for 2 h and reoxygenation with acetate (1 mM) for 24 h. Cell viability was evaluated (*n* = 5). Representative immunoblot images (**G**) and quantification (**H**) of H3K9ac of siNC or siACLY-transfected neurons in the presence or absence of TSA (1 μM) after OGD treatment for 1.5 h and reoxygenation for 12 h. Representative immunoblot images (**I**) and quantification (**J**) of H3K27ac of siNC or siACLY-transfected neurons in the presence or absence of TSA after OGD/R. **K** The primary cultured cortical neurons were subjected to OGD treatment for 2 h and reoxygenation with TSA (1 μM) for 24 h. Cell viability was evaluated (*n* = 5). Data are expressed as means ± S.D., for all panels: *P < 0.05, **P < 0.01, ***P < 0.001, ns stands for no significance by two-way-ANOVA analysis with Bonferroni Test (**A**, **C**, **E**) applied afterward and one-way-ANOVA analysis with Tukey’s Multiple Comparison Test (**F**, **H**, **J**, **K**) applied afterward.

Given that ACLY is a nucleocytoplasmic enzyme capable of catalyzing the generation of acetyl-CoA from acetate, the level of acetyl-CoA in neurons was detected. OGD/R could reduce acetyl-CoA content in neurons, and the level of acetyl-CoA in the siACLY-transfected neurons was even lower (Fig. 5A). Since acetyl-CoA serves as a key substrate for histone acetylation, we next assessed histone acetylation levels. Knockdown of ACLY decreased the acetylation levels of histones H3K9 and H3K27 after OGD/R-treatment, whereas no obvious difference was observed between siNC and siACLY-transfected neurons under normal conditions (Fig. 5B–E), which is consistent with cell viability results in Fig. 3. These results indicate that inhibition of ACLY can reduce the level of histone acetylation in OGD/R-treated neurons.

To determine whether the reduction in histone acetylation contributes to increased neuronal death upon ACLY knockdown, we supplemented neurons with acetate, a precursor for acetyl-CoA synthesis, to restore acetyl-CoA levels [31, 32]. CCK8 results showed that acetate supplementation could improve the viability of siACLY-transfected neurons after OGD/R (Fig. 5F). In addition to boosting acetyl-CoA availability, histone acetylation can also be enhanced by suppressing the activity of histone deacetylases (HDAC). We wonder whether supplementary of HDAC inhibitor trichostatin A (TSA) could rescue ACLY inhibition-induced neuronal death. Indeed, TSA treatment restored histone acetylation levels (Fig. 5G–J) and significantly improved the survival of siACLY-transfected neurons following OGD/R (Fig. 5K). Therefore, ACLY-inhibition promotes neuronal death via reducing histone acetylation.

### ACLY-inhibition decreases the expression of mitochondrial complex genes through modulation of histone acetylation

**Fig. 6:**
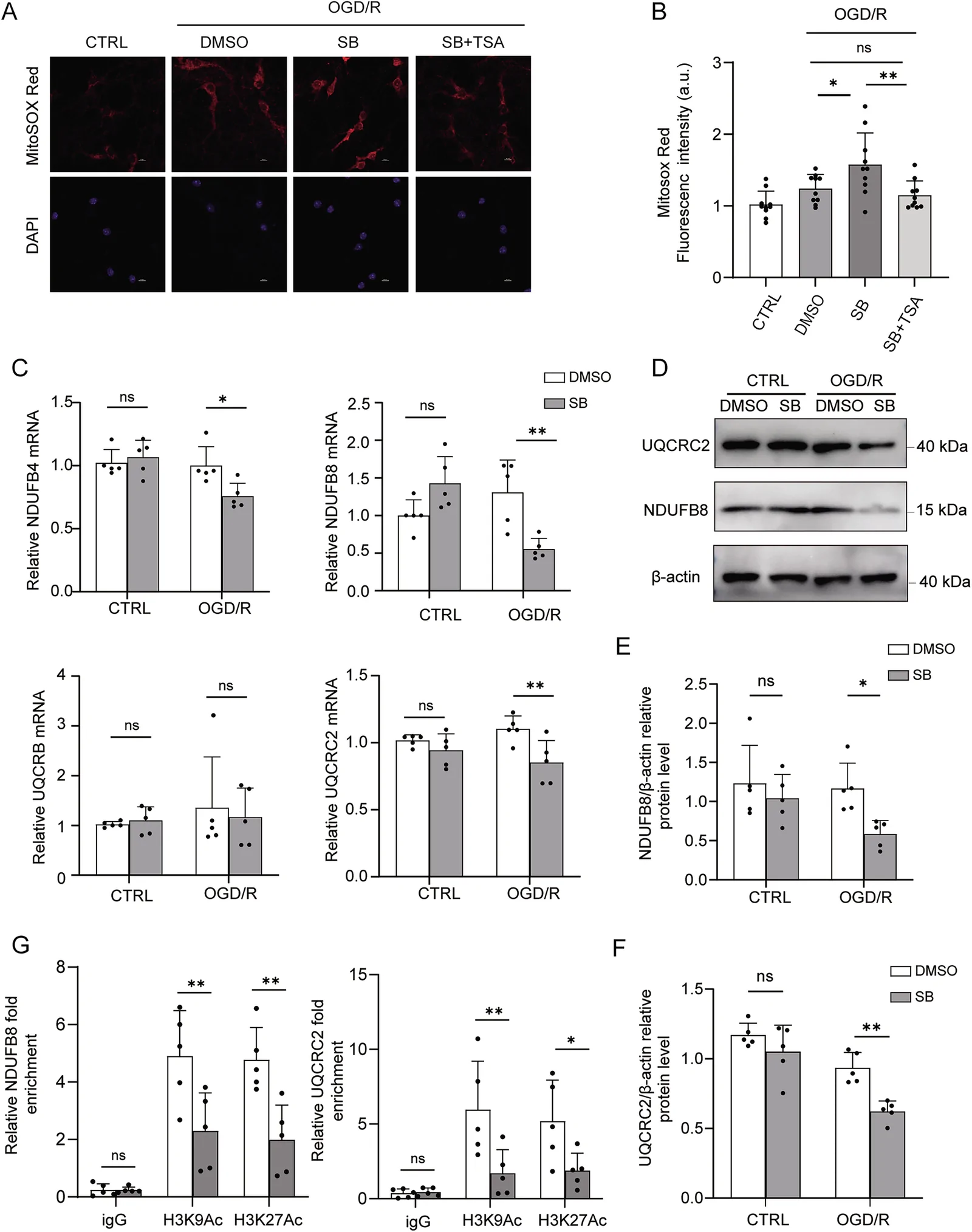
ACLY-inhibition decreases the expression of mitochondrial complex genes via histone acetylation. Representative MitoSOX immunofluorescence staining (**A**) and statistical analysis (**B**) of primary cortical Neurons subjected to OGD treatment for 1.5 h and reoxygenation with SB204990 and for 6 h, scale bar = 10 μm (*n* = 5). **C** qRT-PCR analysis of NDUFB4, NDUFB8, UQCRB, UQCRC2 expression in neurons-treated with DMSO or SB204990 after OGD/R stimulation for 12 h (*n* = 5). Immunoblot images (**D**) and quantification of UQCRC2 (**E**), NDUFB8 (**F**) in neuron-transfected with siNC or siACLY and exposed to OGD treatment for 1.5 h and reoxygenation for 12 h, *n* = 5. **G** CHIP detection of NDUFB8, UQCRC2 acetylation levels in neuronal cells exposed to OGD treatment and reoxygenation with SB204990 for 6 h (*n* = 5). Data are expressed as means ± S.D., for all panels: *P < 0.05, **P < 0.01, ***P < 0.001, ns stands for no significance by two-way-ANOVA analysis with Bonferroni Test (**C**, **E**, **F**, **G**) applied afterward and one-way-ANOVA analysis with Tukey’s Multiple Comparison Test (**B**) applied afterward.

We further explored whether ACLY regulates mitochondria ROS production via histone acetylation. Supplementary of acetate or TSA in ACLY-knockdown neurons reduces mitochondria ROS accumulation after OGD/R (Fig. 6A, B) demonstrating that ACLY can regulate mitochondrial ROS levels by modulating the level of histone acetylation in neurons.

To explore how ACLY regulates mitochondrial ROS in a histone acetylation manner, we referred to our RNA-seq results in Fig. 4A. Genes related to oxidative phosphorylation and ROS production are mainly clustered in mitochondrial complexes (Table S1). Give that mitochondrial complexes I and III are the main sites of ROS production in mitochondria according to previous studies [33], we examined the expression of mitochondrial complex genes after ACLY inhibition. qRT-qPCR results showed that SB204990(SB) decreased the mRNA levels of mitochondrial complex I genes (NDUFB4 and NDUFB8), and complex 3 genes (UQCRC2) in neurons after OGD/R (Fig. 6C). Consistent with the RT-qPCR results, the protein levels of UQCRC2 and NDUFB8 were also reduced (Fig. 6D–F). Furthermore, ChIP assay demonstrated that ACLY inhibition diminished the enrichment of active histone marks H3K9ac and H3K27ac at the promoter regions of NDUFB8 and UQCRC2, instead of the promoters of NDUFB4 and UQCRB (Fig. 6G, Fig. S2A). Correspondingly, the level of acetyl-CoA was also reduced after ACLY inhibition (Fig. S2B). Therefore, ACLY-inhibition decreases the expression of mitochondrial complex genes by reducing histone acetylation.

### MitoTEMPO or TSA administration attenuates ACLY inhibition-induced I/R injury

**Fig. 7:**
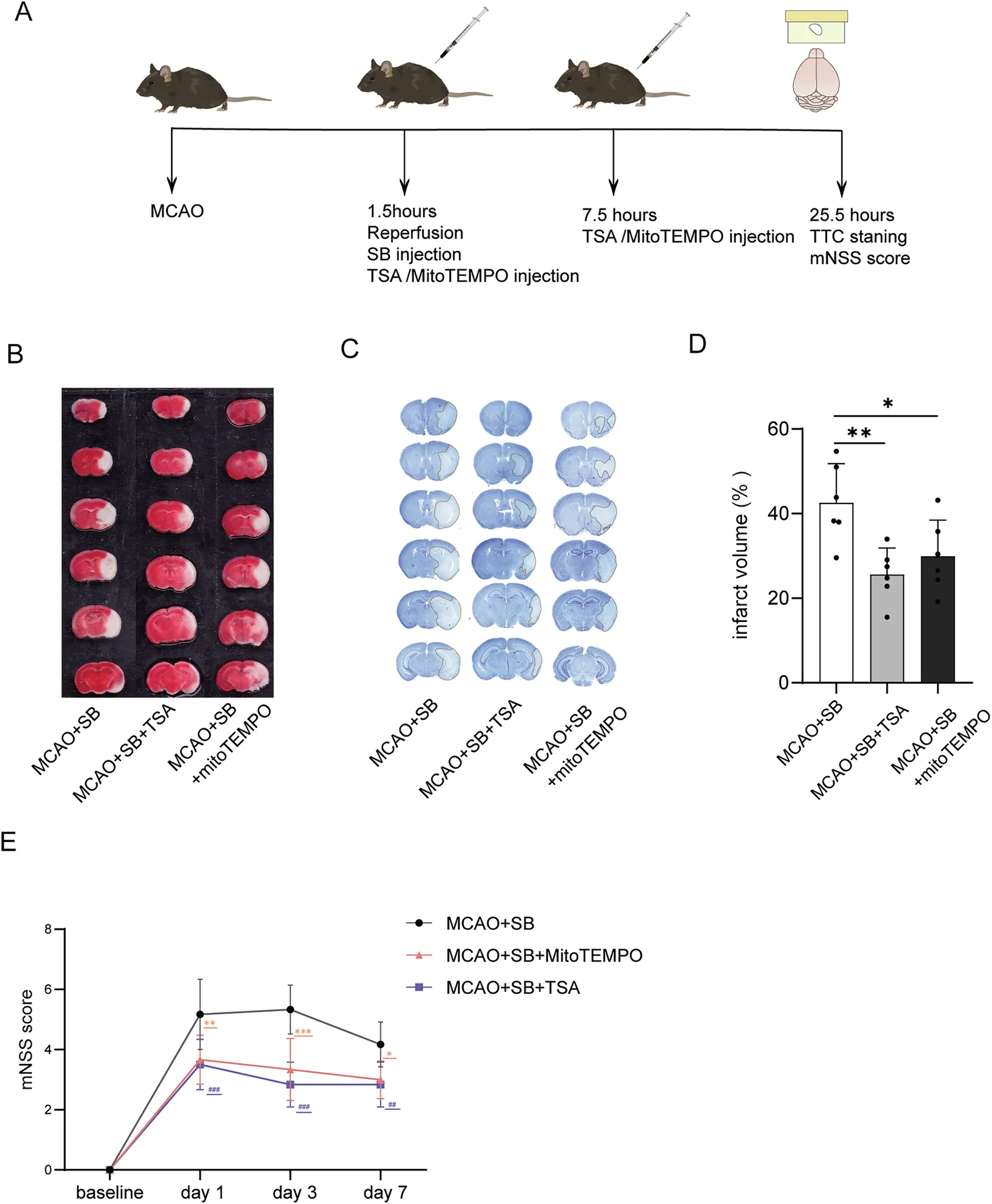
MitoTEMPO or TSA administration attenuates ACLY-inhibition-induced I/R injury. **A** Schematic diagram of the detail relevant operational procedure from tMCAO to TTC staining. **B** Representative brain slices stained by TTC of brain infarct volume at 24 h after tMCAO (*n* = 6). Representative brain slices stained by Nissl (**C**) and quantification (**D**) of infarct volume at 24 h after tMCAO (*n* = 6). **E** Evaluation of neurological deficits at day 1, day 3 and day 7 following reperfusion (*n* = 6). Data are expressed as means ± S.D., for all panels: *P < 0.05, **P < 0.01, ***P < 0.001, ^##^*P* < 0.01, ^###^*P* < 0.001 compared to MCAO + SB by one-way-ANOVA analysis with Tukey’s Multiple Comparison Test (**D**) and two-way-ANOVA analysis with Bonferroni Test (**E**).

Given that mitoTEMPO and TSA can reduce ROS production and neuronal death in vitro, we next investigated whether administration of these two compounds could alleviate brain injury induced by ACLY inhibition in vivo. Immediately after reperfusion following tMCAO, SB204990 was administered simultaneously with either vehicle, mitoTEMPO or TSA and then evaluated brain infarction and neurological deficit scores (Fig. 7A). As predicted, co-administration of TSA or mitoTEMPO respectively with SB204990 reduced the infarction volume induced by ACLY inhibition (Fig. 7B–D, Fig. S3A). Consistently, neurological function was also improved following the combined administration of TSA or mitoTEMPO with SB204990 at day 1, 3 and 7 after MCAO (Fig. 7E). These data collectively indicates that ACLY can alleviate cerebral I/R injury by regulating histone acetylation and mitochondrial ROS levels.

## Discussion

Cell metabolism is implicated in the pathogenesis of cerebral I/R injury [34]. Currently, how lipid metabolism regulates cerebral I/R injury remains largely unknown. Our findings reveal a novel metabolic-epigenetic axis mediated by ACLY in the regulation of cerebral I/R injury.

Aberrant activity or expression of ACLY disrupts metabolic homeostasis and contributes to the pathogenesis and progression of various diseases such as liver I/R injury, non-alcoholic fatty liver disease (NAFLD) and embryo development and cardiac fibrosis [18, 23, 35, 36]. Most of the studies found that ACLY exerts their biological functions primarily through the acetyl-CoA–histone acetylation axis [37]; however, emerging evidence also demonstrates that ACLY mediates the acetylation of non-histone proteins, such as FSP1, thereby regulating cellular susceptibility to ferroptosis [38]. Notably, ACLY itself can undergo acetylation, which influences the development of NAFLD [39]. Additionally, ACLY can induce lipid metabolism remodeling and impair mitochondrial function to exert its regulatory effects [40, 41]. In this study, we found that ACLY expression is upregulated in damaged neurons during cerebral I/R injury and ACLY inhibition enhanced neuronal death and exacerbated cerebral I/R injury via acetyl-CoA-histone acetylation axis. Collectively, these findings expand our understanding of the mechanistic roles of ACLY in disease pathogenesis and highlight its potential as a therapeutic target.

Histone acetylation, a crucial epigenetic modification, is dynamically regulated by histone acetyltransferases (HATs) and histone deacetylases (HDACs) [42]. It modulates transcriptional activity by regulating chromatin structure relaxation [43]. Accumulating evidence highlights the critical involvement of histone acetylation in ischemic stroke pathophysiology. After ischemic brain injury, the balance between histone acetylation and deacetylation is altered [44]. Notably, inhibition of HDAC activity has been shown to exert neuroprotective effects through multiple mechanisms, including activation of the transcription factor Nrf2 [45, 46], suppression of inflammatory responses [47], regulation of the fusion efficiency of autophagosomes and lysosomes [48], and enhancement of the acetylation levels on both histones and non-histones [42]. In the present study, we found that inhibiting the activity of ACLY after OGD/R reduced the abundance of acetyl-CoA, the essential substrate used by HATs for acetylating histone lysine residues. Consistent with this funding, exogenous supplementation of acetate, a metabolic precursor for acetyl-CoA, restored neuronal viability after OGD/R. Further mechanistic investigation revealed that ACLY inhibition decreased histone acetylation at H3K9 and H3K27 specifically at the promoters of mitochondrial complex genes. Importantly, treatment with TSA, a selective HDAC inhibitor, not only reversed the reduction in histone acetylation caused by ACLY inhibition but also improved neuronal survival and reduced infarct volume in a tMCAO model. These findings suggest that metabolic shifts following cerebral ischemia-reperfusion injury can influence gene expression by altering histone epigenetic modifications. In particular, the ACLY–acetyl-CoA–histone acetylation axis may serve as a regulatory mechanism affecting mitochondrial function via modulation of nuclear-encoded mitochondrial genes. However, the precise mechanisms by which histone acetylation regulates the expression of specific mitochondrial complex genes warrant further exploration.

Mitochondria serve as the core of energy metabolism in brain tissues [49]. During the ischemic phase, the limited availability of glucose and oxygen impair mitochondrial OXPHOS, leading to a rapid decline in ATP synthesis [50, 51]. Following reperfusion, mitochondrial function fails to recover immediately; instead, metabolic fluctuations exacerbate neuronal damage [52, 53]. In our study, we found that inhibition or knockdown of ACLY after OGD/R significantly increased mitochondrial ROS production, reduced ATP generation, and elevated mitochondrial membrane potential in neurons. RNA-sequencing analysis revealed that genes positively correlated with ACLY expression are predominantly involved in mitochondrial energy metabolism pathways, including oxidative phosphorylation (OXPHOS) and ROS production. Among these, mitochondrial complex proteins, such as NDUFB4, NDUFB8 and UQCRC2, the component of mitochondrial Complex I and III, are significantly regulated by ACLY inhibition-induced histone acetylation changes. Importantly, treatment with the mitochondrial-targeted antioxidant mitoTEMPO partially restored neuronal viability after ACLY inhibition, highlighting the critical role of mtROS in mediating neuronal damage. This finding not only indicates mitochondrial dysfunction on cerebral I/R injury but also explains the potential metabolic cause of increased mtROS production.

In conclusion, our findings demonstrate that ACLY exerts neuroprotective effects following cerebral I/R injury by activating the acetyl-CoA–histone acetylation axis, thereby reducing oxidative stress and improving mitochondrial function. These results suggest that targeting ACLY may represent a promising therapeutic strategy for alleviating oxidative damage and protecting against cerebral I/R injury.

## Supplementary information

## Data Availability

The data generated or analyzed in this study are included in this published article. RNA-seq data has been deposited in the National Center for Biotechnology Information database under access code PRJNA1335777.
