# Supplementary material for: ACLY alleviates cerebral ischemia/reperfusion injury by reducing oxidative stress and enhancing mitochondrial function via histone acetylation
Source: Cell Death Dis. 2026 Jun 22;17(1):772. doi: 10.1038/s41419-026-09021-4 (PMC13538641; doi:10.1038/s41419-026-09021-4)

Supplementary Information

The supplementary information includes Materials and Methods, Supplementary Figures and Figure legends.

Supplementary Materials and Methods

2.1. Neurological Severity Score Analysis

Neurological deficits severity were evaluated using the modified Neurological Severity Score (mNSS) on a 9-point scale, as follows: (1) no neurological dysfunctions (0 point); (2) left forelimb flexion or inability to fully extend right forepaw when suspended by its tail (1 point); (3) Tail suspension led to left shoulder adduction (2 points);(4) Diminished resistance when the animal was pushed laterally toward the left side (3 points); (5) spontaneous movement across all directions but leftward circling only when the tail was pilled (4 points); (6) Spontaneous circling or walking restricted to the left direction (5 points); (7)movement occurring solely in response to stimulation (6 points); (8) absence of response to stimulation (7 points); and (9) mortality associated with stroke (8 points)(1).

2.2. Primary cell culture and OGD treatment

Primary cortical neurons were obtained from the cerebral cortex of embryonic day 16 to 19 (E16-19) of C57BL/6 mice. Briefly, cerebral cortices were dissected, enzymatically dissociated with 0.025% trypsin, and mechanically triturated to prepare a single-cell suspension， then plated it onto poly-D-lysine-coated culture plates and cultured in maintenance medium with 1% B-27 supplement and 2 mM GlutaMAX).

The culture medium was refreshed at 3-day intervals to maintain optimal nutrient status. After 10 days, mature neuronal cultures were used for subsequent experiments. To establish OGD treatment, the medium was replaced with glucose-free DMEM medium for a 90 min culture period in an anaerobic chamber (Baker Ruskinn, UK; 95% N_2 / 5% CO_2) at 37°C. Thereafter, the glucose-free DMEM medium was substituted with complete maintenance medium and neurons were then transferred to a normoxic environment (95% O2 / 5% CO_2) at 37°C for reperfusion (2)

2.3. Antibodies and Reagents

Antibodies from Abclonal were used: ACLY(A3719), β-actin (AC038), Histone H3(A22348); Acetyl-Histone H3-K27(A2771), Acetyl-Histone H3-K9 (A21107) ; Map2 (67015-1-Ig) was purchased from proteintech; NeuN (ABT-5313) was purchased from PTM BIO; HRP Conjugated Goat anti-Rabbit IgG(HA1001), Secondary anti-bodies used for immunochemistry: iFluor™ 488 Conjugated Goat anti-mouse IgG(HA1125), iFluor™ 555 Conjugated Goat Anti-Rabbit IgG (HA1135) were acquired from HUA BIO; SB204990(T16861) was purchased from TargetMol; MitoSox Red was purchased from Invitrogen; TMRE(C2001S), Enhanced ATP Assay Kit（S0027, Nissl Staining Solution (C0117)was purchased from beyotime.

2.4. Quantitative Real-Time PCR (qRT-PCR) Analysis

Total RNA was extracted from neurons using the RNAfast200 purification kit (Fastagen) with the manufacturer's instructions, and reverse-transcribed into cDNA with StarScript Pro All-in-one RT Mix with gDNA Remover (GenStar). qRT-PCR was conducted on a BioRad 96 touch machine using 2×RealStar Fast SYBR qPCR Mix

(GenStar)(3). GAPDH served as the internal contral. The primer sequences for the qRT-PCR analysis of the target genes are provided below: mouse NDUFB4 forward primer:5’-CGGGTTCCAAGTATAAGCCTG-3’; reverse primer:5’-GTTTGGGGTC GTTGTACTGAAG-3’; NDUFB8:forward primer:5’-TGTTGCCGGGGTCATATCC TA-3’; reverse primer:5’-GCATCGGGTAGTCGCCATAC-3’; NDUFA12 forward primer:5’-GGCCTCCTACGGGTTTTCTTC-3’; reverse primer:5’-GGTGGTGTAGA TGACCCATCG-3’; UQCRB forward primer:5’-GGCCGATCTGCTGTTTCAG-3’; reverse primer:5’-TGATGCCTCATAGTCAGGTCC-3’; UQCRC2 forward primer:5’-AAAGTTGCCCCGAAGGTTAAA-3’; reverse primer:5’-CAGAGAAGCAATCAC CAAACCA-3’; mt-ATP6 forward primer:5’-TTGCCCACTTCCTTCCACAA-3’; reverse primer:5’-AGGAGGGTGAATACGTAGGCT-3’; mt-CO1 forward primer:5’-GCTAGCCGCAGGCATTACTA-3’; reverse primer:5’-CTCCGTGTAGGGTTGCAA GT-3’; GAPDH forward primer:5’-TGGTGAAGGTCGGTGTGAACGG-3’; reverse primer:5’-ACTGTGCCGTTGAATTTGCCG-3’.

2.5. Assay of acetyl-CoA contents

To measure acetyl-CoA contents, cells were harvested in PBS buffer and then were homogenized. The resulting lysate was subjected to ultracentrifugation at 120,000 × g for 5 min at 4°C to obtain a clarified supernatant. Acetyl-CoA levels in the supernatant were then quantified using a commercial acetyl-CoA assay kit (E-EL-0125; Elabscience, China) in strict accordance with the manufacturer's protocol(4) Acetyl-CoA concentrations were calculated by referencing the standard curve.

2.6. Immunoblot Analysis

To lyse brains or cells, RIPA buffer supplemented with 1% PMSF and 1% protease/phosphatase inhibitor cocktail was used. Protein concentrations were determined via Bradford assay. Equal amounts of protein samples were separated using 10% SDS-PAGE and transferred onto PVDF membranes. Membranes were blocked with 5% skim milk in TBS-T buffer for 2 h at room temperature (RT), followed by incubation with primary antibodies at 4°C overnight. After washing, membranes were incubated with secondary antibodies for 1 h at RT. Protein signals were visualized by chemiluminescence using an imaging system (Image Quant 800, Cytiva), followed by densitometric densitometric analysis.

2.7. Chromatin Immunoprecipitation Assay (CHIP)

The CHIP kit was purchased from Beyotime. After formaldehyde fixation, cells were collected and ultrasonically fragmented into 200-bp DNA fragments. Subsequently, anti-H3K27ac antibody, anti-H3K9ac antibody or IgG control was added and left to incubate at 4°C overnight. After capturing the immune complexes with Protein A/G agarose beads, the complexes were eluted with eluent and crosslinks were reversed. The DNA was purified by phenol-chloroform extraction and used for qPCR analysis(5). The primer sequences for the promoter regions of target genes were obtained from PrimerBank and their specificity was verified by BLAST.

2.8. Immunohistology and quantification

Brain tissues were fixed in 4% paraformaldehyde (P1110, Solarbio) at 4°C for 12 hours, then transferred them to cryoprotection in 30% sucrose-PBS solution over 48 h. Sucrose-infused tissues were embedded in optimal cutting temperature (OCT)

compound and coronally sectioned into 30 μm thickness slices. Frozen slices were permeabilized using 0.3% Triton X-100 in PBS for 30 min at RT, then were blocked with 10% normal goat serum in PBS for 2 h. Sections were incubated with primary antibodies against ACLY (1:100) and NeuN (1:1000) at 4°C overnight. Subsequently, sections were incubated with secondary antibodies (1:1000) for 1 h at RT. DAPI (1 μg/mL) was used for nuclear counterstaining, and a confocal microscope (Nikon Ti2-E) was employed to capture immunofluorescence signals. Fluorescence intensity was quantified via ImageJ. Cultured neurons were fixed with 4% PFA for 10 min, then were permeabilized with 0.5% Triton X-100 for 10 min, and blocked with 5% normal goat serum in PBS for 2 h at RT. The subsequent immunostaining steps were identical to those described for tissue sections.

2.9. RNA Sequencing and analysis

RNA was extracted from neural cells treated with the SB204990 and DMSO for 12 hours after OGD/R using Trizol reagent (Thermo Fisher Scientific, Germany). The concentration and purity of the extracted RNA were detected using a Nanodrop 2000. RNA integrity was examined via agarose gel electrophoresis, and the RNA Integrity Number (RIN) was determined with an Agilent 2100. Magnetic beads conjugated with Oligo (dT) were used to isolate mRNA from total RNA through A-T base pairing with the polyA tail at the 3' end. Subsequently, library construction and RNA sequencing (RNAseq) were performed using the Truseq™ RNA Sample Prep Kit and the Illumina Novaseq 6000 platform. All the above processes were performed at Cosmos Wisdom

Biotech. The RNA-seq data have been deposited in the National Center for Biotechnology Information database under accession code PRJNA869334.

References

1. Chen HZ, Guo S, Li ZZ, Lu Y, Jiang DS, Zhang R, et al. A critical role for interferon regulatory factor 9 in cerebral ischemic stroke. The Journal of neuroscience : the official journal of the Society for Neuroscience. 2014;34(36):11897-912.

2. Qin C, Yang S, Chu YH, Zhang H, Pang XW, Chen L, et al. Correction To: Signaling pathways involved in ischemic stroke: molecular mechanisms and therapeutic interventions. Signal transduction and targeted therapy. 2022;7(1):278.

3. Cui Y, Zhang Y, Zhao X, Shao L, Liu G, Sun C, et al. ACSL4 exacerbates ischemic stroke by promoting ferroptosis-induced brain injury and neuroinflammation. Brain, behavior, and immunity. 2021;93:312-21.

4. Chen Y, Huang L, Yu T, Zhao M, Zhou J, Wang L, et al. Tailoring Escherichia coli for high-yield production of O-acetyl-L-homoserine through multi-node metabolic regulation. Metabolic engineering. 2025;91:455-65.

5. Lv M, Duan Z, Tan J, Liu J, Wang Q, Wang C, et al. PHGDH-mediated serine synthesis in astrocytes supports neuroinflammation by sustaining NADH level to promote histone acetylation. Cell death & disease. 2025;16(1):397.

Supplementary Table 1.

Genes enriched in SB-treated neural cells in the following two pathways after OGD/R treatment.

Pathway	pvalue	Genes
Oxidative phosphorylation	3.68302161905854e-10	COX2/Ndufv3/Ndufa12/CYTB/Ndufb4/Ndufb4c/Ndufb4b/ND3/COX1/ND2/Uqcrb/ATP6
Chemical carcinogenesis - reactive oxygen species	1.10231962432634e-08	COX2/Ndufv3/Ndufa12/CYTB/Ndufb4/Ndufb4c/Ndufb4b/ND3/COX1/ND2/Gstm2/Uqcrb/ATP6

Differentially expressed genes in neural cells from the SB group and DMSO group were detected using DESeq2 with the screening criteria of |Fold Change| > 1.2 and p value < 0.05, followed by KEGG pathway enrichment analysis using cluster Profiler.

Supplementary Figure Legends

Supplementary Figure 1. ACLY inhibition exacerbates brain ischemia–reperfusion injury.
(A) Quantification of infarct volume at 24 h after tMCAO, (n=6 for each treatment). Data are expressed as means ± S.D., for all panels: **P <0.01, ***P < 0.001 by one-way-ANOVA analysis with Tukey's Multiple Comparison Test applied afterward.

Supplementary Figure 2. ACLY-inhibition decreases the expression of mitochondrial complex genes via histone acetylation.
(A)CHIP-qPCR analysis of H3K9Ac and H3K27Ac enrichment on promoters of NDUFB4 and UQCRB in neuronal cells exposed to OGD treatment and reoxygenation with SB204990 for 6 h (n=5). (B) ELISA analysis of acetyl-CoA levels in the supernatants from neuronal cells-treated with DMSO or SB204990 followed by OGD/R treatment (n=5). Data are expressed as means ± S.D., for all panels: ns stands for no significance by two-way-ANOVA analysis with Bonferroni Test.

Supplementary Figure 3. MitoTEMPO or TSA administration attenuates ACLY-inhibition-induced I/R injury.
(A)Quantification of infarct volume at 24 h after tMCAO, (n=7 for each treatment). Data are expressed as means ± S.D., for all panels: **P <0.01, ***P < 0.001 by one-way-ANOVA analysis with Tukey's Multiple Comparison Test.

Supplementary Figure 1

A

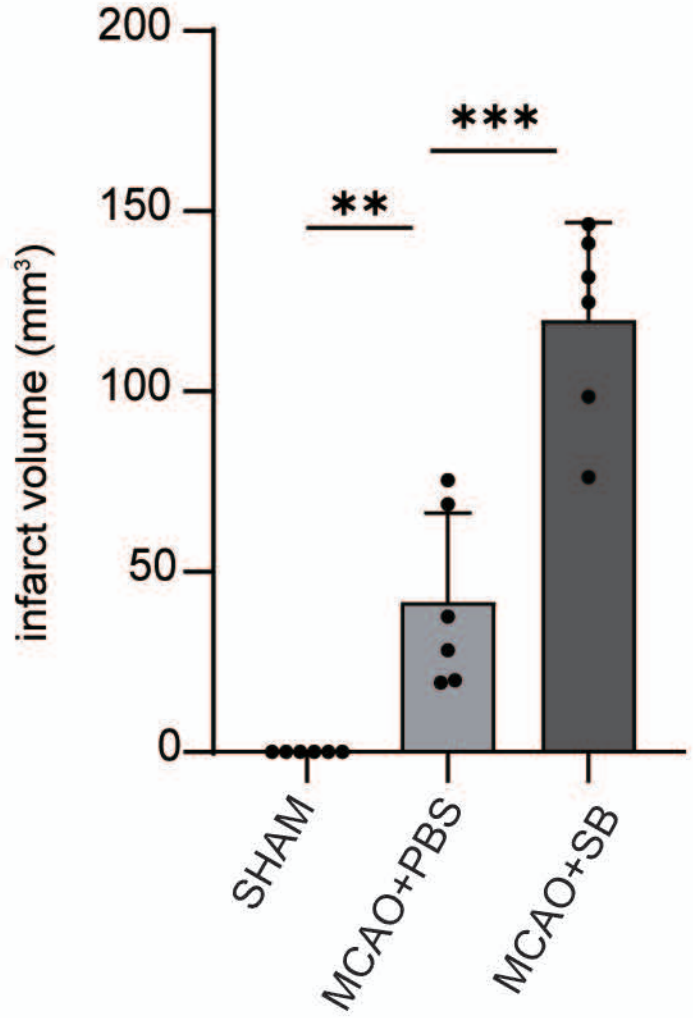

Supplementary Figure 2

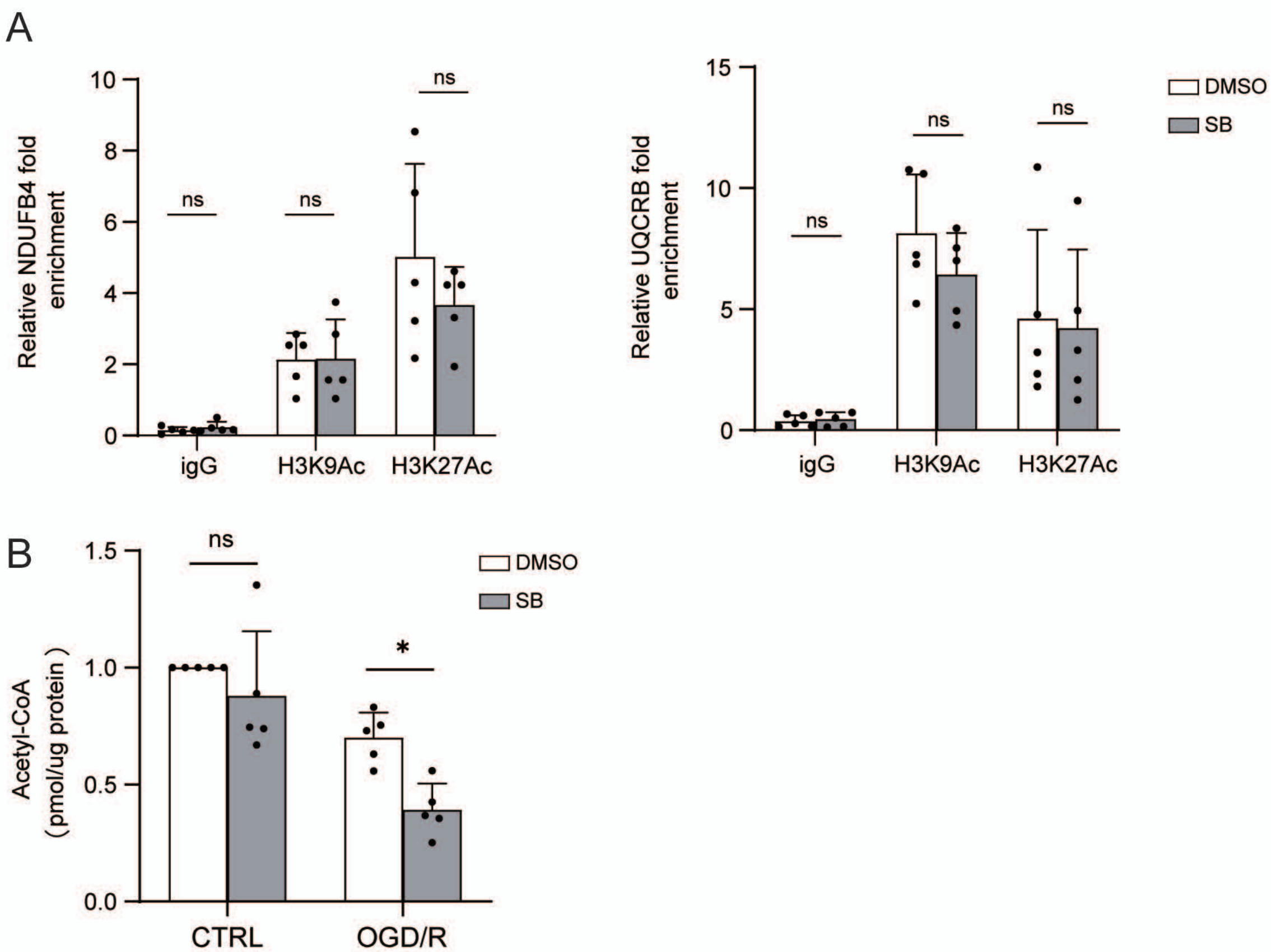

Supplementary Figure 3

A

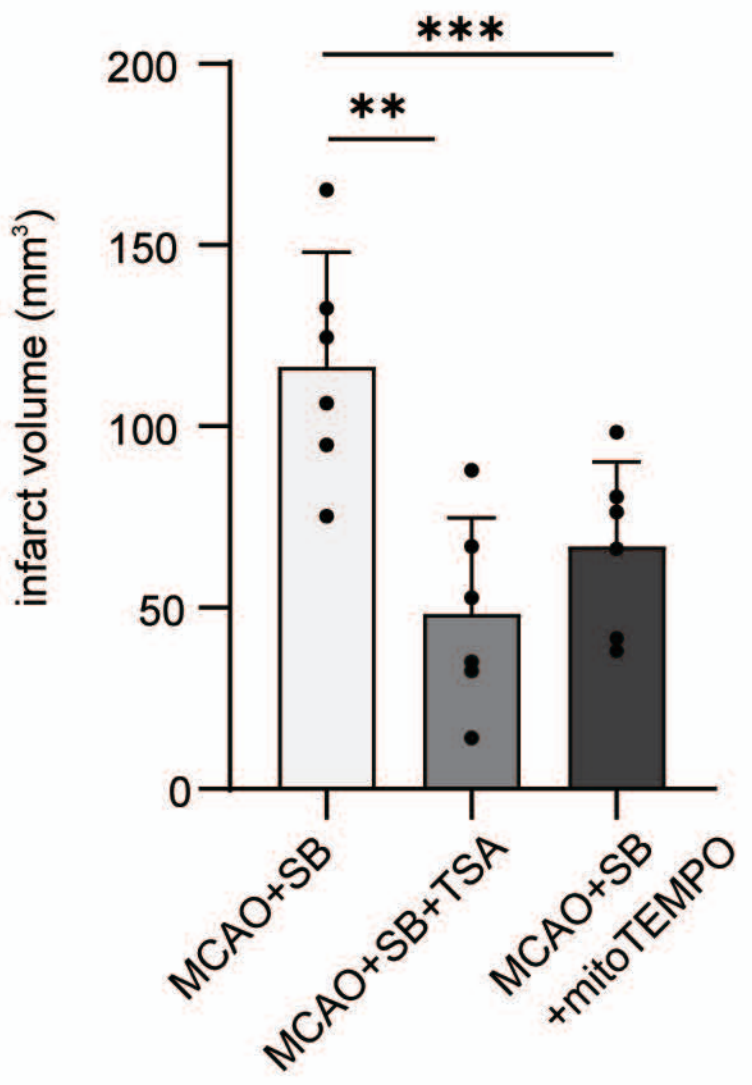

Supplement: Supplementary file 1 [file 41419_2026_9021_MOESM2_ESM.pdf]
